# Characterization of Molecular Mechanisms Controlling *fabAB* Transcription in *Pseudomonas aeruginosa*

**DOI:** 10.1371/journal.pone.0045646

**Published:** 2012-10-02

**Authors:** Herbert P. Schweizer, Kyoung-Hee Choi

**Affiliations:** 1 Department of Microbiology, Immunology, and Pathology, IDRC at Foothills Campus, Colorado State University, Fort Collins, Colorado, United States of America; 2 Department of Oral Microbiology, College of Dentistry, Wonkwang University, Iksan, Chonbuk, South Korea; Vrije Universiteit Brussel, Belgium

## Abstract

**Background:**

The FabAB pathway is one of the unsaturated fatty acid (UFA) synthesis pathways for *Pseudomonas aeruginosa*. It was previously noted that this operon was upregulated in biofilms and repressed by exogenous UFAs. Deletion of a 30 nt *fabA* upstream sequence, which is conserved in *P. aeruginosa*, *P. putida*, and *P. syringae*, led to a significant decrease in *fabA* transcription, suggesting positive regulation by an unknown positive regulatory mechanism.

**Methods/Principal Findings:**

Here, genetic and biochemical approaches were employed to identify a potential *fabAB* activator. Deletion of candidate genes such as *PA1611* or *PA1627* was performed to determine if any of these gene products act as a *fabAB* activator. However, none of these genes were involved in the regulation of *fabAB* transcription. Use of *mariner*-based random mutagenesis to screen for *fabA* activator(s) showed that several genes encoding unknown functions, *rpoN* and DesA may be involved in *fabA* regulation, but probably via indirect mechanisms. Biochemical attempts performed did fail to isolate an activator of *fabAB* operon.

**Conclusion/Significance:**

The data suggest that *fabA* expression might not be regulated by protein-binding, but by a distinct mechanism such as a regulatory RNA-based mechanism.

## Introduction

The common opportunistic human pathogen, *Pseudomonas aeruginosa*, causes serious infections in immunocompromised or cystic fibrosis patients. Fatty acid synthesis is essential for cellular function by providing metabolic precursors for synthesis of many cellular components. The fatty acid biosynthetic enzymes are therefore potential targets for new antibacterials. It was previously established that the biosynthesis of fatty acids in *P. aeruginosa* closely resembles the pathway established in *Escherichia coli*, and consists of two phases, initiation and elongation (reviewed in [1]). Besides exhibiting dehydratase activity, FabA also catalyzes the isomerization of *trans* fatty acids produced during *de novo* synthesis of fatty acids containing a *cis*-double bond. These UFAs are then condensed with malony-ACP by FabB, thus bypassing the FabI (enoyl-ACP reductase)-catalyzed step and maintaining the double bond. In subsequent rounds of elongation, full-length UFAs are formed [2]. This pathway is the main route for producing the 16:1^Δ9^- and 18:1^Δ11^-ACP thioesters that are incorporated into phospholipids by the glycerol-phosphate and acyl-glycerol-phosphate acyltransferases (PlsB and PlsC) [3].

Since UFAs are required for maintaining the fluidity of bacterial membranes, numerous studies on UFAs have been performed in many prokaryotes, especially *E. coli.* However, the pathways of UFA synthesis remained mostly uncharacterized in *P. aeruginosa*.

In a recent study, it was demonstrated that UFA biosynthesis in *P. aeruginosa* can occur via three different pathways depending on the availability of oxygen: 1) the aerobic pathways encoded by the fatty acid desaturase genes *desA* and *desBC* [3], and 2) anaerobic pathway encoded by the *fabAB* operon [2]. It was shown that a Δ*fabA* mutant required UFA supplementation during anaerobic growth, but not during aerobic growth, demonstrating that although the aerobic pathway supports UFA formation, the *fabAB* operon is indispensable for the UFA synthesis under anaerobic conditions [3].

In *E. coli*, two distinct transcriptional regulators regulate expression of the *fabA* and *fabB* genes involved in UFA synthesis. FadR, which negatively regulates expression of the *fad* genes involved in fatty acid transport and β-oxidation [4], also acts as a positive transcriptional regulator of *fabA* and *fabB* gene expression [5], [6]. It was shown that an *E. coli fadR* mutant synthesized less UFAs compared to wild-type strains and that *fabA*(Ts) *fadR* double mutants required UFA supplementation for growth, suggesting that FadR also functions as an activator of UFA synthesis [7]. In addition, *fabA* activation by FadR is repressed by exogenous long acyl-CoAs by binding to the FadR protein [6], [8]. Furthermore, *fabB* was also shown to be positively controlled by FadR because *fadR* mutants exhibit cerulenin hypersensitivity, and show conditional lethal phenotypes in *fadR fabB* double mutants [5]. In contrast, FabR negatively controls UFA synthesis in *E. coli* by binding to similar sequences in the *fabA* and *fabB* upstream regions [9]. Although a FabR homologue PA4890 showing 50% similarity to the *E. coli* protein was found in *P. aeruginosa*, it seems to play only a minor role, if any, in *fabA* regulation [3].

Previous searches failed to identify a *P. aeruginosa* FadR homolog responsible for activation of *fabAB* transcription in *P. aeruginosa* [10] and thus, the regulation of UFA synthesis in *P. aeruginosa* seems distinct from *E. coli.* Even though the activity of FabA and FabB from *P. aeruginosa* is very similar to that of the same enzymes from *E. coli*, there is one obvious distinction: whereas the *E. coli fabA* and *fabB* genes are located in two different regions of the chromosome, the *P. aeruginosa fabAB* genes form an operon [1], [2]. The different organization of these genes may imply the existence of distinct regulatory pathways of UFA biosynthesis in *P. aeruginosa*.

Here, we demonstrate that *P. aeruginosa fabAB* operon expression seems to be controlled by a complex regulatory network composed of a combination of directly acting factors and indirectly acting transcriptional or translational regulators and other metabolic factors, rather than transcriptional regulators directly acting in the *fabAB* regulatory region.

## Materials and Methods

### Bacterial strains, plasmids, media and culture conditions

The bacterial strains and plasmids in this study are listed in Table 1. *E. coli* and *P. aeruginosa* strains were maintained on Luria-Bertani medium (LB; 10 g per liter tryptone, 5 g per liter yeast extract, 10 g per liter NaCl; Becton, Dickinson & Co., Sparks, MD). Since *P. aeruginosa* cells are able to utilize citrate as a sole carbon source and energy source but not *E. coli*, citrate-based VBMM medium (Vogel-Bonner Minimal Medium; 3.0 g Na_3_Citrate [citric acid Na_3_ salt], 2.0 g Citric acid [free acid], 10.0 g K_2_HPO_4, 3.5 g_ NaNH_4_PO_4_×4 H_2_O, 1 mM MgSO_4_×7 H_2_O, and 0.1 mM CaCl_2_) was used for counter-selection against *E. coli* after biparental matings between *E. coli* and *P. aeruginosa*. For plasmid maintenance in *E. coli*, the media were supplemented with 100 µg/ml ampicillin, 25 µg/ml chloramphenicol, 35 µg/ml kanamycin or 15 µg/ml gentamycin. For marker selection in *P. aeruginosa*, 200 µg/ml carbenicillin, 30 µg/ml of gentamycin and 150 µg/ml of spectinomycin was used, as appropriate. If necessary, the monounsaturated fatty acid oleic acid (OA) was added together with 0.05% Brij-58 to solubilize the fatty acid at a final concentration of 0.05%.

**Table 1:** Bacterial strains and plasmids used in this study.

Strains or plasmids		Relevant properties	Reference or source
*E. coli*			
SM10*lacI^q^*		*thi thr leu tonA lacY supE recA::*RP4-2-Tc::Mu Km *lacI^q^*	T. Hoang
SM10 (λ*pir*)		*thi thr leu tonA lacY supE recA::*RP4-2-Tc::Mu Km λ*pir*	[15]
DL291		F- *araD139* Δ(*argF-lac*)*U169 rpsL150 deoC1 relA1 rbsR ptsF25 flbB5301 glpR2 gyrA* Δ(*glpT-glpA*)593 *recA1*	[18]
DL291-Tn*7* b		Gm^r^; DL291 derivative containing mini-Tn*7*T-*fabA′-′lacZY* gene fusion	This study
*P. aeruginosa*			
PAO1		Prototroph	[36]
PAO434		PAO1 with mini-Tn*7*T-*lacZ*	This study
PAO435		PAO1 with mini-Tn*7*T-p*fabA′-lacZ* a resulted from integration of pPS1460	This study
PAO459		PAO1 with mini-Tn7T-p*fabA′-lacZ* resulted from integration of pPS1491	This study
PAO460		PAO1 with mini-Tn7T-p*fabAΔ30′-lacZ*	This study
PAO483		PAO434 with Δ*PA4890::FRT* a	This study
PAO484		PAO435 with Δ*PA4890::FRT*	This study
PAO491		PAO434 with Δ*PA1539::FRT*	This study
PAO492		PAO435 with Δ*PA1539::FRT*	This study
PAO495		PAO460 with Δ*PA4890::FRT*	This study
PAO497	PAO460 with Δ*PA1539::FRT*		This study
PAO517	PAO435 with Δ*PA4890::FRT* Δ*PA1539::FRT*		This study
PAO194	PAO1 with temperature-sensitive *fabA*(Ts)		This study
PAO1010	PAO435 with Δ*anr*::*FRT*		This study
PAO1151	Gm^ra^; PAO1 with mini-Tn*7*T-p*PA1612*′-*lacZ*		This study
Plasmid			
pPS752	Ap^r^; *fabA^+^ fabB* ^+^ (4.8 kb chromosomal *Bam*HI-*Eco*RI fragment cloned between the same sites of pUC18)		[2]
pPS790	Ap^r^; *fabA^+^ fabB* ^+^ (2,039 bp PCR amplified *Bam*HI-*Kpn*I fragment from pPS752 cloned between the same sites of pUC19)		[2]
pPS1450	Ap^r^, Gm^r^; mini-Tn*7* delivery vector		[37]
pPS1453	Ap^r^, Gm^r^; mini-Tn*7* delivery vector with a promoter-less *lacZ* gene		[37]
pPS1460	Ap^r^, Gm^r^; ligation of ∼300 bp *Sal*I-*Sma*I fragment from pPS790 to *Xho*I-*Nru*I fragment of pPS1453		This study
pPS1482	Ap^r^; pCR2.1 with PCR-amplified *PA1611-fabA* intergenic region from PAO1		This study
pPS1488^b^	Ap^r^; pCR-Blunt II-TOPO with PCR-amplified *PA1611-fabA* intergenic containing a 30 bp deletion		This study
pPS1491	Ap^r^, Gm^r^; pUC18-mini-Tn*7*T-p*fabA*′-*lacZ* delivery vector obtained by ligation of ∼230 bp *Kpn*I-*Sma*I fragment of pPS1482 into the same sites of pPS1453		This study
pPS1492	Ap^r^, Gm^r^; pUC18-mini-Tn*7*T-p*fabAΔ30*::*lacZ* delivery vector obtained by ligation of ∼230 bp *Kpn*I-*Sma*I fragment of pPS1488 into the same sites of pPS1453		This study
pDONR221	Km^r^; Gateway donor vector		Invitrogen
pEX18Ap	Ap^r^; gene replacement vector with MCS from pUC18		[13]
pEX18ApGW	Ap^r^; Gateway destination vector		[14]
pPS1669	Ap^r^, Gm^r^; pUC18-mini-Tn*7*T-Gm-*lacZ*-GW		[14]
pFLP2	Ap^r^; source for Flp recombinase		[13]
pPS856	Ap^r^, Gm^r^; Gm-*FRT* cassette		[13]
pBT20	Ap^r^, Gm^r^; *mariner* transposon delivery vector		S. Lory
pPS1472	Ap^r^, Km^r^; pCR2.1 with amplified *PA1611* from PAO1		This study
pPS1476	Ap^r^; pEX18Ap-*PA1611* (ligation of ∼2.0 kb blunt-ended *Eco*RI fragment of pPS1472 to *Pst*I+*Eco*RI cleaved and blunt-ended pEX18Ap)		This study
pPS1479	Ap^r^, Gm^r^; pEX18Ap-Δ*PA1611*::Gm (ligation of blunt-ended *Kpn*I-*Nco*I fragment of pPS1476 to ∼1.1 kb *Sma*I Gm^r^::*FRT* fragment of pPS856)		This study
pPS1503^b^	Ap^r^, Gm^r^; pEX18Ap-Δ*PA1627*::Gm (ligation of the *Stu*I-*Xho*I fragment of PCR-amplified Δ*PA1627*::Gm from PAO1 into the *Sma*I-*Sal*I sites of pEX18Ap)		This study
pPS1506^b^	Ap^r^, Gm^r^; pEX18Ap-Δ*PA1539*::Gm (ligation of the *Msc*I-*Xho*I fragment of PCR-amplified Δ*PA1539*::Gm into the *Sal*I-*Sma*I sites of pEX18Ap)		This study
pPS1505^b^	Ap^r^, Gm^r^; pEX18Ap-Δ*PA4890*::Gm (ligation of the *Xho*I-*Sma*I fragment of PCR amplified Δ*PA4890*::Gm into *Sal*I-*Sma*I sites of pEX18Ap		This study
pPS1634	Ap^r^, Gm^r^; pUC18R6K-mini-Tn*7*T-Gm-p*fabA::lacZY* (ligation of three fragments, *Bam*HI-*Xho*I fragment of pPS1633, ∼250 bp *Bam*HI-*Eco*RV fragment of pPS790 and ∼6.2 kb *Sma*I-*Sal*I fragment of pMC1403 into pPS1633)		This study
pPS1633	Ap^r^, Gm^r^; pUC18R6K-mini-Tn*7*T-Gm		[37]
pMC1403	Ap^r^; *lacZY* fusion cloning vector for construction of translational fusions		[17]
pPS1733	Ap^r^, Km^r^ ; *anr* ^+^ (ligation of 0.8 kb PCR fragment amplified with Anr-UP-KpnI and Anr-DN-BamHI to pCR2.1 vector)		This study
pPS1682	Sp^ra^; *anr* ^+^ (ligation of 0.8 kb *Kpn*I-*Bam*HI fragment of pPS1733 into *Kpn*I-*Bam*HI cleaved pVLT35)		This study
pPS1684	Ap^r^; *anr* ^+^ (ligation of 0.8 kb *Kpn*I-*Bam*HI fragment of pPS1733 into *Kpn*I-*Bam*HI cleaved pUCP20)		This study
pPS1991^b^	Ap^r^, Gm^r^; pPS1669 with *att*L1-p*PA1612*-*att*L2 to promote *lacZ* expression		This study

a Abbreviations: Ap, ampicillin; *att*, λ attachment site (s); *FRT*, Flp recombinase target site; Gm, gentamycin; Km, kanamycin; MCS, multiple cloning site; p, promoters; Sp, spectinomycin

b see text for plasmid or strain construction details.

### DNA manipulations and vector constructions

Routine procedures were employed for manipulation of DNA [11]. Plasmid DNA was isolated using the QIAprep Mini-spin kit (Qiagen, Valencia, CA) and *P. aeruginosa* chromosomal DNA was isolated using the QIAamp DNA Mini Kit (Qiagen, Valencia, CA). DNA fragments were purified from agarose gels utilizing the QIAquick gel extraction kit (Qiagen, Valencia, CA).

The *lacZ* fusion plasmid containing a 30 bp deletion within the *fabA* upstream region was created in several steps. The *fabA* upstream region was PCR-amplified from PAO1 genomic DNA using primers IR187-UP and IR187-DN (sequences of primers used in this study are listed in Table 2.). These primers introduced *Sma*I and *Sal*I restriction enzyme cleavage sites at each end of the PCR fragment. The PCR fragment was purified and ligated into the TA cloning vector pCR2.1 (Invitrogen), resulting in pPS1482. This plasmid was used as a template in a two-step PCR reaction to generate pPS1488 containing a 30 bp deletion in the *fabA* upstream region. In the first PCR reaction, two flanking DNA segments harboring overlapping sequences were amplified using two sets of primers (PCRSOE-A+IR187SOE-B) and (IR187SOE-C+PCRSOE-D). In the second PCR, each purified 1^st^ round PCR fragment was used to perform splicing by overlap extension (SOE) PCR using primers PCRSOE-A and PCRSOE-D. The resulting PCR product was cloned into pCR-Blunt II-TOPO vector (Invitrogen), resulting in a plasmid containing the *fabA* upstream sequences with a 30 bp deletion. Finally, pPS1491 and pPS1492 were constructed by ligating the *Kpn*I-*Sma*I fragments of pPS1482 and pPS1488, respectively, into pPS1453 cleaved with the same enzymes.

**Table 2:** Primers used in this study.

Primer Name	Sequence (5′ → 3′)
*fabA* upstream primers	
pfabA0	cgggaatgaacgattacctg
pfabA-attB1^a^	GGGGACAAGTTTGTACAAAAAAGCAGGCTcatgaccggatcgccttcgaa
pfabA1-attB2	GGGGACCACTTTGTACAAGAAAGCTGGGTtctacgacaagggcggcaagc
pfabA2-attB2	GGGGACCACTTTGTACAAGAAAGCTGGGTggacgcgggaataaagtgaac
pfabA3-attB2	GGGGACCACTTTGTACAAGAAAGCTGGGTatctgttcgccggacactgtg
pfabA4-attB2	GGGGACCACTTTGTACAAGAAAGCTGGGTactttcaccgcaacgcaacag
pfabA4a-attB2	GGGGACCACTTTGTACAAGAAAGCTGGGTctgtgactttcaccgcaacg
pfabA5-attB2	GGGGACCACTTTGTACAAGAAAGCTGGGTtctatgactaggctgccgctg
pfabA6-attB2	GGGGACCACTTTGTACAAGAAAGCTGGGTcgacgccgatacaataacccg
pfabA7-attB2	GGGGACCACTTTGTACAAGAAAGCTGGGTgcgcgacggccgctggacgaa
pfabA8-attB2	GGGGACCACTTTGTACAAGAAAGCTGGGTccgccacaaccctgcagttca
pfabA9-attB2	GGGGACCACTTTGTACAAGAAAGCTGGGTgggatttttgaggagctcgc
pfabA10-attB2	GGGGACCACTTTGTACAAGAAAGCTGGGTatgaccaaacaacacgccttc
pfabA11-attB2	GGGGACCACTTTGTACAAGAAAGCTGGGTatcagcgatgtcggcggcaag
pPA1612-UP-attB2	GGGGACCACTTTGTACAAGAAAGCTGGGTcgccacctgctctacttca
*fabA* Δ30	
PCRSOE-A	AGGTATCCGGTAAGCGGCAG
IR187SOE-B	TGTCCGGCGAACAGATGTTC
IR187SOE-C	GAACATCTGTTCGCCGGACATGACTAGGCTGCCGCTGCGA
PCRSOE-D	CGGTTCCTTTAGCAGCCCTT
*PA1627* deletion	
1627SOE-A	gtgatcagttgcagcatcaccg
1627SOE-B	TCAGAGCGCTTTTGAAGCTAATTCGacccgggacagacccatgact
1627SOE-C	AGGAACTTCAAGATCCCCAATTCGggaagaaacatccaatcatcggat
1627SOE-D	accagcagtactaccaggaacc
*PA4890* deletion	
4890SOE-A	tgaaggattccgtctgcaagcc
4890SOE-B	TCAGAGCGCTTTTGAAGCTAATTCGgaggacatacggcttcctttgg
4890SOE-C	AGGAACTTCAAGATCCCCAATTCGgctgcgtttcatcatgatcggc
4890SOE-D	tagttgaactccgcctcgccat
*PA1539* deletion	
1539SOE-A	gcagggtagtagttgtgcgaca
1539SOE-B	TCAGAGCGCTTTTGAAGCTAATTCGtcgctcatgttccacctggttg
1539SOE-C	AGGAACTTCAAGATCCCCAATTCGcgagtgacagctcgatgtcctt
1539SOE-D	cgactaccgtttctgaatccgc
*anr* deletion	
anr-UpF-GWL	TACAAAAAAGCAGGCTttgacagggtgcgacaggta
anr-UpR-Gm	TCAGAGCGCTTTTGAAGCTAATTCGaatccttgcagtgtgcttgg
anr-DwnF-Gm	AGGAACTTCAAGATCCCCAATTCGggaagtgcacatcctcgact
anr-DwnR-GWR	TACAAGAAAGCTGGGTacgaagctgtccacggtcat
Nested PCR primers	
Rnd1-TnM	TATAATGTGTGGAATTGTGAGCGG
Rnd1-Pa1	GGCCACGCGTCGACTAGTACNNNNNNNNNNGATAT
Rnd2-TnM	ACAGGAAACAGGACTCTAGAGG
Rnd2-Pa	GGCCACGCGTCGACTAGTAC
TnMSeq	CACCCAGCTTTCTTGTACAC
TnMRev	TGCACCGTGCAGTCGATGATAA
Other primers	
IR187-UP	GAGGGGAcCCGGgATGATCTACGACAA
IR187-DN	AGGTCTTCTCGGGTaccGGCGTGTTGT
Gm-UP	TGGAGCAGCAACGATGTTAC
Gm-DN	TGTTAGGTGGCGGTACTTGG
FabU	GGGATCCGGAATGATCTACGACAAGG
FabD	GGGTACCAAGTTTAGCCCGTTCATGC
PA1611-UP	AAATCCCTGAACTGCAGGGTTGTG
PA1611-DN-in	ACCTGCGCCAAGAATTCACCCATA
GmFRT-UP	CGAATTAGCTTCAAAAGCGCTCTGA
GmFRT-DN	CGAATTGGGGATCTTGAAGTTCCT
Anr-UP-KpnI	ATCGCGGCTGCGGTACCCTT
Anr-DN-BamHI	ATACAACGGATCCGCGCTGAG
EcglmS-DN	TGCAGCTGCTGGCTTACCATG
Tn*7*R	CACAGCATAACTGGACTGATTTC
DIG-fabA	DigN-ATGCGATCGATCATCAGCATGTTG
Bio-fadR-for	Biotin-GGACACTGTGACTTTCACCGCAACGCAACAGTCTATGACT
fadR-Rev	AGTCATAGACTGTTGCGTTGCGGTGAAAGTCACAGTGTCC
fadR-UP	GAACCGGACACTGTGACTTTCACCGCAACGCA-ACAGTCTATGACTA
fadR-DN	TAGTCATAGACTGTTGCGTTGCGG-TGAAAGTCACAGTGTCCG
30-concatamer-for	(TAGACTGTTGCGTTGCGGTGAAAGTCACAG)_3_
30-biotin-rev	Biotin-CTGTGACTTTCACCGCAACGC

a Sequences in capital letters are common for all genes amplified in a particular experiment and overlap with the sequences of the other genes that they were spliced to, e.g. Gm or *attB* primer sequences. Lower-case letters indicate gene-specific sequences.

For promoter localization experiments, 11 different portions of the *fabA* intergenic sequence were fused to a promoter-less *lacZ* contained on a mini-Tn*7* delivery vector utilizing Gateway universal cloning technology (Invitrogen) as described in Choi et al., 2006. The *fabA* upstream region was amplified using 11 different primer pairs consisting of combinations of pfabA-attB1 and pfabA1-attB2 to pfabA11-attB2, giving PCR segments containing progressively shorter lengths of the *fabA* upstream region at 20∼21 bp intervals. To construct a *lacZ* fusion with the *PA1612* promoter, a fragment containing the *PA1612* promoter, the *PA1612*-*PA1611* operon, and the entire *PA1611-fabA* intergenic region was also amplified by PCR using the primer pair pfabA-attB1 and pPA1612-UP-attB2. The individual PCR fragments thus obtained contain *att*B1 and *att*B2 sites which facilitate high-throughput cloning into the universal donor vector pDONR221 (Invitrogen) containing *att*P1 and *att*P2 sites by a site-specific recombination between *att*B and *att*P sites, resulting in plasmids harboring different inserts of *fabA* upstream sequences with flanking hybrid *att* sites, *att*L1 and *att*L2. This was followed by a second site-specific recombination between the *att*L and *att*R sites of the resulting plasmids and the destination vector pPS1669, respectively, resulting in mini-Tn*7* elements containing transcriptional *lacZ* fusions with different lengths of *fabA* upstream sequences.

### Random *mariner*-based insertional mutagenesis

*mariner*-based random mutagenesis of PAO1 containing a temperature-sensitive *fabA* allele (PAO1 *fabA*(Ts)) [2] was performed by electroporating pBT20 [12] containing a *mariner* transposon with a Gm-resistance marker. It was reasoned that knock-out of potential *fabAB* activators would create unconditional mutants requiring OA supplementation at all temperatures. Identification of OA auxotrophs was performed by patching Gm^r^ transformants on LB containing 30 µg/ml gentamycin with and without OA. Chromosomal fragments from OA auxotrophs were transferred into the PAO1 *fabA*(Ts) parental strain containing a chromosomally integrated *fabA*′-*lacZ* fusion to assess whether the mutation generated by *mariner* transposon insertion directly affected *fabAB* expression. β-galactosidase assays were performed in cells grown in the presence of OA.

*mariner*-based random mutagenesis was also performed in PAO1-*fabA*(Ts) strain containing a chromosomally integrated *fabA*′-*lacZ* fusion. A knock-out of a potential activator would create an unconditional mutant requiring OA supplementation at all temperatures and result in lighter blue colonies. Lighter blue colonies were tested for OA auxotrophy by patching Gm^r^ transposon mutants on LB containing 30 µg/ml gentamycin with or without OA supplementation. In addition, β-galactosidase assays were performed in cells grown in the presence of OA. The transposon insertion points in the mutants harboring an OA auxotrophy were first determined by PCR amplification using Gm-UP and Gm-DN primers to verify *mariner* transposon insertions in the PAO1 chromosome. Secondly, the absence of insertions in the *fabAB* operon was confirmed by PCR amplification using *fabAB* specific primers FabU and FabD. The insertion sites of transposons located outside of the *fabAB* genes were determined by two-step random PCR amplifications using *mariner* specific primers (Rnd1-TnM and Rnd2-TnM), random PAO1 chromosomal primers (Rnd1-Pa1), and its nested primer Rnd2-Pa. The resulting PCR fragments were sequenced by using primer TnMSeq to determine chromosomal *mariner* transposon insertion sites (S. Lory, personal communication). An alternative method employed to map *mariner* insertion sites was as follows. Chromosomal DNAs from OA auxotrophs were digested with *Bam*HI which cuts the *mariner* transposon once, followed by self-ligation of chromosomal DNA fragments self-ligated and PCR amplification with primers Rnd2-TnM and TnMRev. The resulting PCR fragments were sequenced as above.

### Gene deletion

For construction of a gene replacement vector containing a deletion allele of the *PA1611* gene, this gene was amplified by using primers PA1611-UP and PA1611-DN-in. The PCR fragment was cloned into pCR2.1 to yield pPS1472 followed by the ligation of the ∼2.0 kb blunt-ended *Eco*RI fragment of pPS1472 to *Pst*I+*Eco*RI cleaved and blunt-ended pEX18Ap vector. The *PA1611* coding sequence was deleted by replacement of a blunt-ended *Kpn*I-*Nco*I fragment of pPS1476 with a 1.1 kb *Sma*I Gm^r^::*FRT* fragment of pPS856 [13], resulting in pEX18Ap-Δ*PA1611*::Gm.

The plasmids pPS1503, pPS1505, pPS1506 containing Δ*PA1627*::Gm, Δ*PA4890*::Gm and Δ*PA1539*::Gm, respectively, were constructed by SOEing PCR as described in the following procedure. Five ng of pPS856 plasmid was used for amplification of Gm-*FRT* cassette using primers GmFRT-UP and GmFRT-DN Flanking gene-specific DNA fragments were amplified using the following primer pairs: 1627SOE-A+1627SOE-B and 1627SOE-C+1627SOE-D; 4890SOE-A+4890SOE-B and 4980SOE-C+4890SOE-D; or 1539SOE-A+1539SOE-B and 1539SOE-C+1539SOE-D for *PA1627*, *PA4890*, or *PA1539*, respectively. Since each primer-B and primer-C contains sequences that overlap with the Gm-*FRT* fragment, the resulting three PCR fragments - i.e., the two flanking DNA fragments and the Gm-*FRT* segment - were annealed together and subjected to second round PCR amplification using each primer-A and primer-D. PCR amplification resulted in the generation of a deletion allele of *Gene*::Gm, which was subcloned into the gene replacement vector pEX18Ap [13]. These gene replacement vectors with Δ*PA1611*::Gm, Δ*PA1627*::Gm, Δ*PA4890*::Gm and Δ*PA1539*::Gm were transformed into *E. coli* SM10*lacI^q^* cells and then conjugally transferred to PAO1 and PAO1 harboring mini-Tn*7*T-*lacZ* or the mini-Tn*7*T-*fabA′lacZ* chromosomal fusion by biparental mating for 5 h, followed by plating the recombinants on VBMM plates containing 30 µg/ml of gentamycin. Merodiploid recombinants were purified on the same medium and were resolved by streaking individual Gm^r^ colonies on a LB plate containing 5% sucrose. Mutant constructions were verified by colony PCR, followed by Flp-mediated excision of Gm^r^ marker, which was performed by the conjugal transfer of the Flp-producing pFLP2 into the recombinants [13].

For creation of an *anr* deletion mutant, a deletion allele of Δ*anr*::Gm was created by PCR amplification using two primer pairs of anr-UpF-GWL+anr-UpR-Gm and anr-DwnF-Gm+anr-DwnR-GWR and Gateway cloning technology was utilized as described in Choi et al. [14]. For complementation experiments, *anr* was amplified by using primers Anr-UP-KpnI and Anr-DN-BamHI, the PCR fragment was ligated into pVLT35 [15] or pUCP20 [16], resulting in pPS1682 or pPS1684, respectively. The plasmids were individually transformed into the wild-type and Δ*anr* mutant strain containing a chromosomal p*fabA*′-*lacZ* transcriptional fusion by using the rapid electroporation method described in Choi et al. [10]. Transformants were selected on LB plates containing 150 µg/ml of spectinomycin or 200 µg/ml of carbenicillin for pVLT35 or pUCP20, respectively.

### Construction of an *E. coli* host strain and a genomic library of *P. aeruginosa*

**Figure 1:**
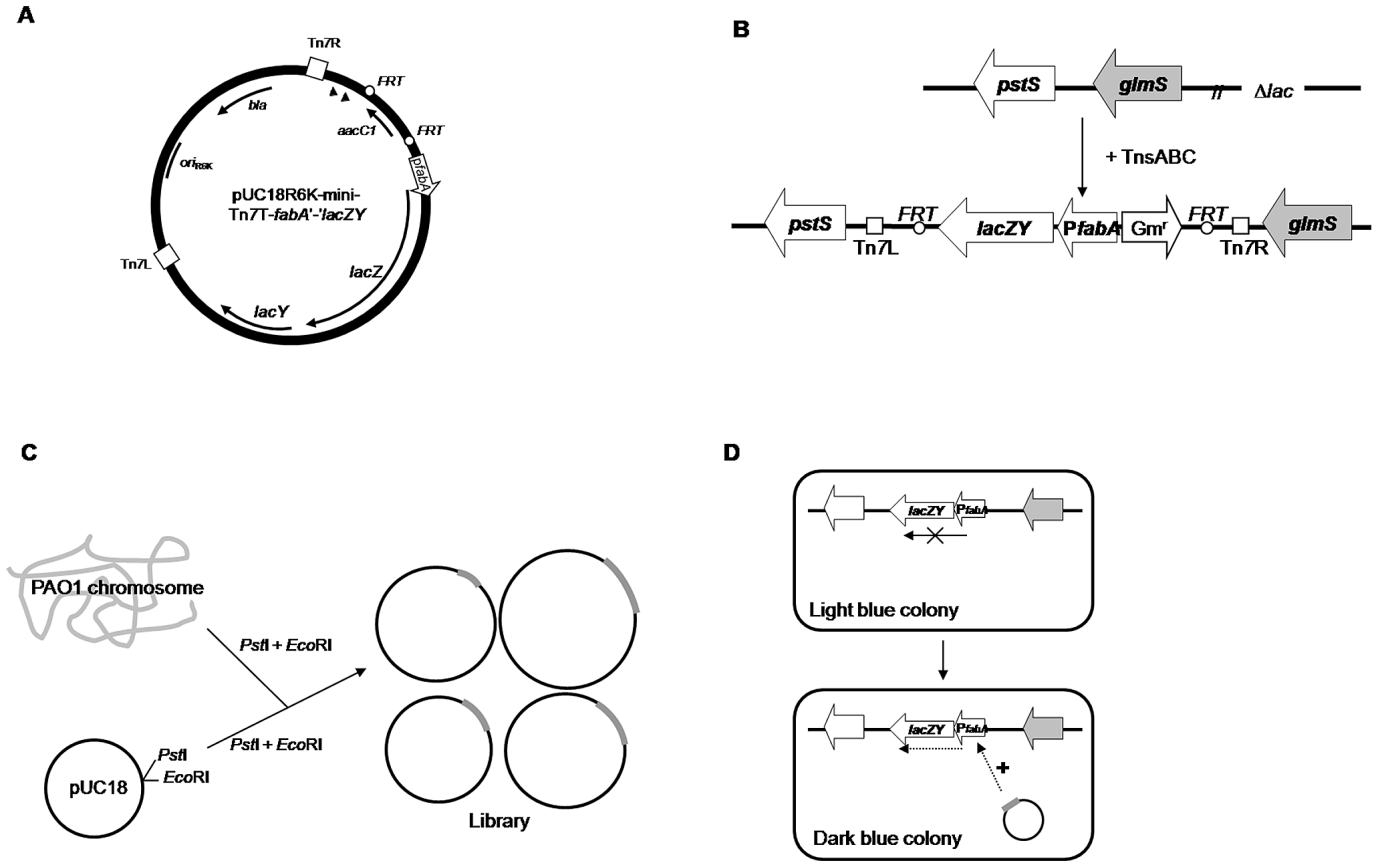
Construction of an *E. coli* host strain and a genomic library of *P. aeruginosa*. **A**) A *fabA*′-′*lacZY* translational fusion was assembled on a mini-Tn*7* suicide delivery vector. **B**) The mini-Tn*7* vector was co-electroporated with the Tn*7* transposase expressing helper plasmid pTNS1 into an *E. coli* Δ*lac* strain. Since the suicide delivery vector cannot replicate in *E. coli* due to the presence of the conditional protein-dependent *ori*R6K, gentamycin-resistant (Gm^r^) transformants will result from site- and orientation-specific integration at the chromosomal *att*Tn*7* site which is located immediately downstream of the *glmS* gene in the *glmS - pstS* intergenic region. **C**) A *Pst*I-*Eco*RI *P. aeruginosa* chromosomal DNA library was constructed by ligation of partially digested *Pst*I-*Eco*RI fragments into pUC18. **D**) The library was used to transform a *P. aeruginosa* strain harboring a chromosomally integrated *fabA*′-′*lacZY* fusion. Since *fabA*′-′*lacZY* is only expressed at low levels, the host strain will only form light blue colonies on X-Gal-containing indicator medium. Transformants expressing putative activating proteins indicated by “+” will appear as darker blue colonies. Abbreviations: Ap^r^, ampicillin resistance; *FRT*, Flp-recombinase target; Tn*7*L and Tn*7*R, left and right end of Tn*7*, respectively.

For identification of an activator of *fabA* expression in a heterologous host, gene fusion technology was employed (Fig. 1). First, the p*fabA*′-′*lacZY* translational gene fusion-containing Tn*7* delivery plasmid pPS1634 was constructed by ligation of three fragments: a *Bam*HI-*Xho*I fragment of pPS1633, an ∼250 bp *Bam*HI-*Eco*RV fragment of pPS790 and an ∼6.2 kb *Sma*I-*Sal*I fragment of pMC1403 [17], resulting in an in-frame fusion of a portion of the *fabA* coding sequence plus *fabA-PA1611* intergenic region to *lacZY* (Fig. 1A). Second, an *E. coli* strain containing a chromosomal insertion of the *fabA′-′lacZY* fusion construct was created by co-electroporating pPS1634 and pTNS1 into the Δ*lac E. coli* strain DL291 [18], resulting in the site-specific integration of *fabA′-lacZY* fusion at *att*Tn*7* downstream of *glmS* in the *E. coli* chromosome. The integration into *att*Tn*7* was verified by PCR amplification using primer pair EcglmS-DN and Tn*7*R (Fig. 1B). Third, a PAO1 library was created by ligation of *Eco*RI+*Pst*I partially-digested chromosomal DNA fragments ranging from 0.75 kb to 5.0 kb into *Pst*I+*Eco*RI-digested pUC18 (Fig. 1C). After transforming the library into *E. coli* containing the *fabA*′-′*lacZY* gene fusion, putative activator-encoding plasmids were selected as dark blue colonies on LB-ampicillin plates containing 40 µg/ml of X-Gal and 25 µM of IPTG (Fig. 1D). Plasmids isolated from blue colonies were retransformed into *endA* ^−^ *E. coli* cells to facilitate recovery of high quality plasmid DNA. Plasmids from isolates showing dark blue color were mapped by digestion with restriction enzymes *Pst*I and *Eco*RI, *Bam*HI, *Hin*dIII or *Sph*I. For the identification of the putative *fabA*-activating gene encoded by these plasmids, vector-chromosomal DNA junction sequences were determined using the M13 forward and reverse sequencing primers.

### β-galactosidase assays

Cells were grown at 37°C with shaking to exponential phase (optical density at 540 nm ∼0.4–0.8) in LB medium with 0.05% Brij 58+/−0.05% oleic acid. β-galactosidase (βGal) activity was measured in chloroform/sodium dodecyl sulfate-permeabilized cells and its activity calculated as previously described [19]. The experiment was performed in triplicate.

### RT-PCR analysis

Total RNA was extracted from PAO1 cells grown at 37°C and 16°C using the hot phenol method [20]. Using 1 µg of RNA, cDNA was synthesized using primer DIG-fabA following the procedure described in the Invitrogen manual with minor modifications. The cDNA was then used as a template for PCR amplification using different primer sets consisting of DIG-fabA, and primers annealing at various positions in the intergenic region, which included pfabA1-attB2 through pfabA9-attB2 or the primer pfabA0 within the *PA1611* coding region. PCR fragments were analyzed by agarose gel electrophoresis.

### Purification of *fabA* activator

First, streptavidin beads were used to purify a FadR-like *fabA* activator A biotinylated, double-stranded DNA oligonucleotide (Bio-fadR-for and fadR-Rev) composed of the 30 bp putative regulatory region and 5 bp flanking sequences was prepared as follows: 1 µg of both oligonucleotides (Bio-fadR-for and fadR-Rev) were denatured by incubation for 5 min at 95°C and then re-annealed either by slow cooling to room temperature or reducing the temperature to 25°C in 2.5°C increments in a thermal cycler. For preparation of crude cell extracts, three liters of *P. aeruginosa* PAO1 cells were grown at 37°C until they reached an optical density (540 nm) of 0.8. Cells were harvested by centrifugation, then passed through a French press three times in 27 ml of buffer (20 mM Tris-HCl [pH 7.9], 0.5 mM NaCl, 10% glycerol and 1 mM PMSF). Cell debris was removed by centrifugation at 12,000×g for 10 min followed by ultracentrifugation at 34,000×g for one hour. The supernatant was concentrated by addition of ammonium sulfate to 60% saturation. The protein precipitate was collected by centrifugation and resuspended in 3 ml of 10 mM Tris-HCl (pH 7.5), 10% glycerol and then dialyzed at 4°C overnight against 3 liters of the same buffer. Three µg of the resulting protein extract was added to a DNA binding reaction composed of 1 µg of poly-dI-dC, 1 µg of dsDNA sequence, and 5× binding buffer (100 mM Hepes [pH 7.6], 5 mM EDTA, 50 mM (NH_4_)_2_SO_4_, 50 mM DTT, 1% Tween 20 and 150 mM KCl). After incubation at room temperature for 15 min, 100 µl of μMACS streptavidin microbeads were added and the mixture was applied to a column which was equilibrated with 100 µl of protein equilibration buffer provided in the kit and rinsed twice with 100 µl of binding buffer in the magnetic separation unit. After sample application, the column was washed six times with binding buffer before DNA-bound proteins were eluted by the addition of binding buffer containing 0.2–1.0 M NaCl. The protein contents of eluted samples were analyzed by 10% SDS-PAGE.

Second, CNBr-activated Sepharose beads were used to purify *fabA* activator according to the procedure described by other research group with minor modification [21]. A duplex of two oligonucleotides (fadR-UP and fadR-DN) was created same as above. Similar to the method described above, dialyzed sample was prepared. The dialyzed mixture was applied to a column packed with CNBr-activated Sepharose beads (Amersham Pharmacia Biotech, Piscataway, NJ). Unbound proteins were removed by washing with three volumes of column buffer. DNA-bound protein(s) were eluted by the addition of TE buffer (10 mM Tris-HCl [pH 7.5], 1 mM EDTA) containing 0.2–0.8 M NaCl. The protein contents in eluted samples were analyzed by electrophoresis on a 10% SDS-PAGE gel. All fractions showing distinct bands were collected and proteins were concentrated with microcon centrifugal filter units (Millipore). Protein bands were cut from the SDS-PAGE gel, subjected to in-gel trypsin digestion, and their identities confirmed using peptide mass fingerprinting.

Third, affinity chromatography was performed using a 30 bp concatamer generated by PCR amplification using 30-concatamer-for and its complementary 30-biotin-rev primer. Then DIG-labeled concatamer was prepared as the manufacturer's manual (Roche Applied Science, Indianapolis, IN). Cell lysates from PAO1 cells grown overnight in 200 ml of LB medium were prepared by cell lysis in a high salt buffer (20 mM Tris-HCl [pH 8.0], 0.5 M NaCl, 1 mM PMSF, 1 mM EDTA, 0.1% Triton X-100 and 10 µg/ml lysozyme) using freeze-thaw cycles. The cell lysates were subjected to ultracentrifugation at 34,000×g for 1 h (Beckman, SWTi28 rotor) followed by dialysis against 4.5 liters of buffer (10 mM Tris-HCl [pH 7.5], 10% glycerol). DNA binding reactions contained 1 µg of poly-L-lysine, 1 µg of poly-dI-dC, binding buffer (20 mM Hepes [pH 7.6], 1 mM EDTA, 10 mM (NH_4_)_2_SO_4_, 1 mM DTT, 0.2% Tween 20 and 30 mM KCl), 10–40 ng of concatamer DNA and 0.2–2 µg of protein extract, and the mixtures were incubated at room temperature for 30 min. The reaction mixtures were loaded onto a 6% native polyacrylamide gel prepared and electrophoresed in 0.5× TBE buffer. DNA fragments were transferred to neutral or positively charged nylon membranes for biotin or DIG detection, respectively. The detection of biotin- or DIG-labeled DNA was performed following the protocols of the NEBlot Phototope detection kit (New England Biolabs, Beverly, MA) or the DIG gel shift kit (Roche Applied Science, Indianapolis, IN), respectively.

### DINAMelt analysis

This was performed on “http://www.bioinfo.rpi.edu/applications/hybrid/hybrid2.php” using the sequences in *fabA* 5′ untranslated region (UTR).

## Results

### Characterization of *fabA* regulatory region

**Figure 2:**
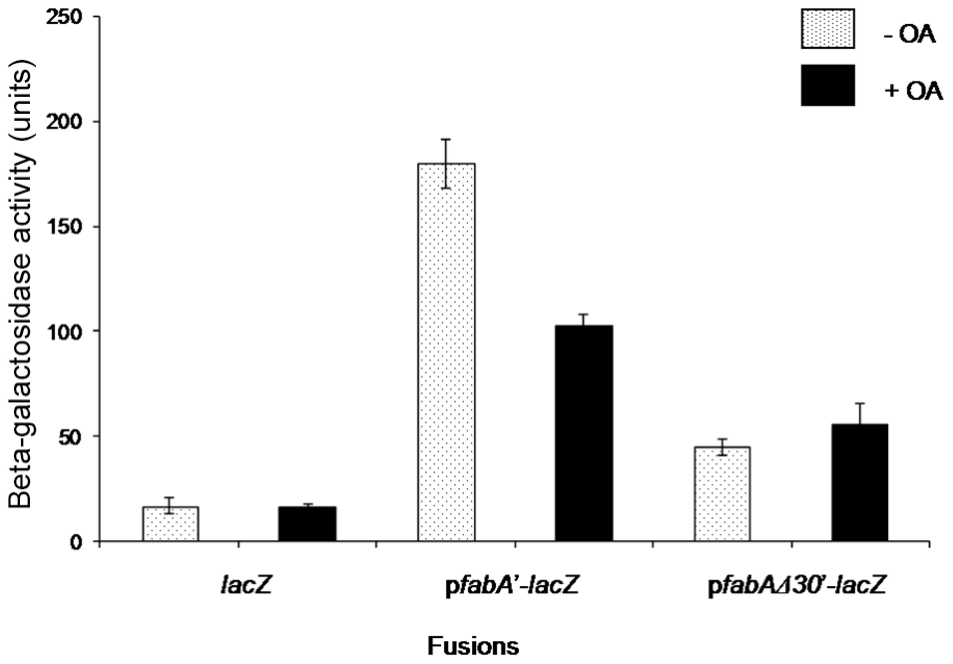
*lacZ* expression in PAO1 containing chromosomally integrated *lacZ, fabA′-lacZ or fabA*Δ30*′-lacZ* transcriptional fusions. Strains were grown to mid-log phase in LB medium with or without oleate (OA) supplementation and β-galactosidase activities were measured. Activities are expressed in Miller Units.

The 188 bp *PA1611-fabA* intergenic region contains several recognizable features. It contains a putative −10 region (TACAAT), but no corresponding −35 region, suggesting a promoter requiring an activator for RNA polymerase binding to initiate *fabAB* transcription (Fig. S1). It also contains a 30 bp sequence which is conserved in all *Pseudomonas* species sequenced to date (Fig. S2). This conservation includes *PA1611* which encodes a probable sensor kinase/response regulator hybrid protein. This strong sequence conservation suggests that the 30 bp sequence may be an important *fabAB* regulatory region. A highly conserved fatty acid biosynthesis repressor (FabR, or DesT in *P. aeruginosa*) binding site was also found upstream of the 30 bp sequence in *P. aeruginosa*, which may be involved in negative regulation of *fabA* expression. The DesT binding site is highly conserved in *fabA* upstream regions of several other bacteria (Fig. S3). We hypothesized that *fabA* regulation may be complex and mediated by yet unknown regulatory protein(s) which act at conserved upstream regions. However, the organization of the *fabA* upstream regions of *E. coli* and *P. aeruginosa* is different. Whereas the *E. coli* FabR repressor binds at a site overlapping the −10 region and the FadR activator binds at a site overlapping the −40 region, *P. aeruginosa* contains a putative DesT repressor-binding site upstream of the 30 bp putative activator-binding site (Fig. S4). To assess whether the 30 bp sequence plays a role in *fabA* regulation, it was deleted and the corresponding region was fused to *lacZ*. After single copy insertion into the PAO1 chromosome, βGal activities were measured in the absence or presence of OA and compared to that expressed in the wild-type *fabA*′-*lacZ* fusion strain. As shown in Fig. 2, OA repressed *fabAB* expression in the wild-type strain harboring a chromosomal *fabA′*-*lacZ* fusion, whereas *fabA*Δ30*′-lacZ* expression was significantly decreased compared to wild-type *fabA′-lacZ*. In addition, *fabAB* expression in the *fabA*Δ30 deletion construct was no longer repressible in the presence of OA. These results suggested that the 30 bp sequence is indeed involved in regulation of *fabA* expression and may be a binding site for an unknown activator or other effector molecule.

**Figure 3:**
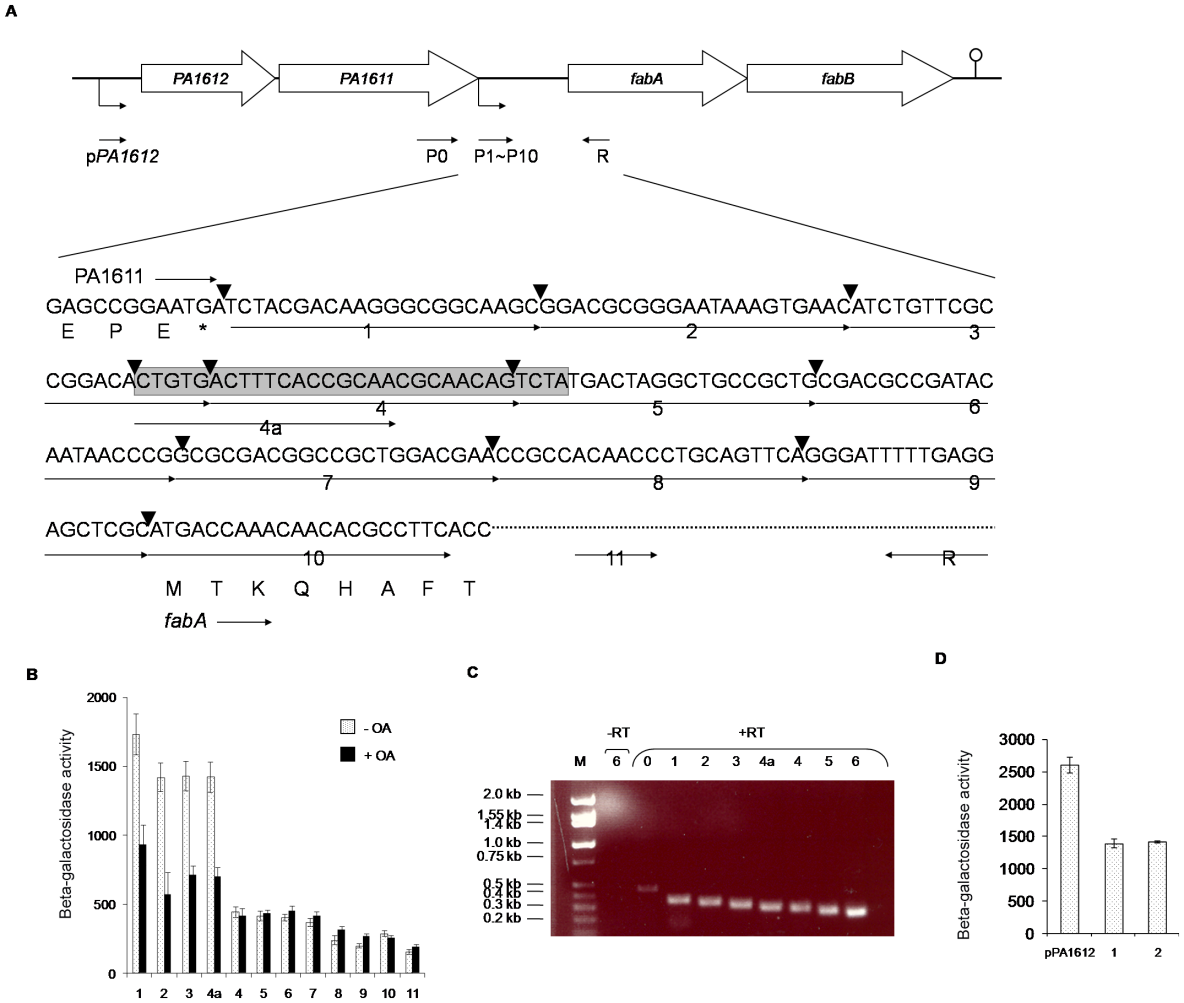
Characterization of *fabA* regulatory region. **A**) Positions of primer-binding sites in the *fabA-PA1611* intergenic region. Each primer is symbolized as follows: P0, fabA0; P1-P11, fabA1-attB2 through fabA11-attB2; R, fabA-attB1. Primer P11 is placed at the 124^th^–144^th^ nucleotide from the first nucleotide of the *fabA* coding region. The sequence shaded and boxed in the gray box indicates the putative 30 bp regulatory element. Vertical arrow heads indicate the end points of sequences present in the *lacZ* fusion constructs analyzed in Fig. 3B. **B**) The 30 bp sequence is important for *fabAB* expression. PAO1 contained *fabA′-lacZ* vectors with *fabA* upstream regions amplified with primers 1 through 11. The 5 bp addition in primer 4a, which restores a complete 30 bp sequence, recovered *lacZ* expression indicating that it is important for *fabA* transcription. Cells were grown and β-galactosidase activities were measured as described in the legend to Fig. 2 with and without oleate (OA) supplementation. **C**) Characterization of the promoter region of *fabA* using RT-PCR analysis of *fabA* expression. RNA was extracted from PAO1 grown at 37°C using the hot phenol extraction method. cDNA was synthesized using the Superscript III First-strand kit (Invitrogen) and primer R. Resulting cDNAs were used as templates for PCR amplification utilizing primer R and the indicated primers (see Fig. 3A for location of primer-binding sites). **D**) β-galactosidase activities in PAO1 containing *lacZ* fusions with various *fabA* upstream fragments. The upstream fragments were amplified with primers pPA1612, and primers p1 and p2 (see Fig. 3A for primer-binding sites).

This was confirmed by analyses using *lacZ* transcriptional fusions containing progressively shorter *fabA* upstream fragments (Fig. 3A). The data shown in Fig. 3B demonstrate that deletion of the 30 bp sequence and beyond led to loss of *lacZ* activity and OA responsiveness. The 5 bp addition in primer 4a, which restores a complete 30 bp sequence, recovered *lacZ* expression indicating that this sequence is important for *fabA* transcription (Fig. 3B).

In order to further define the promoter region of *fabA*, RT- PCR was performed. RNA extracted from wild-type PAO1 cells grown at 37°C was reverse-transcribed to cDNAs, which were then used as templates for PCR amplification utilizing primer R and the primers indicated in Fig. 3A. PCR products were apparent in all reactions containing either primer 0 which primes within the *PA1611* coding region, or primers 1 through 6 which prime in the *fabA* intergenic region (Fig. 3C; see Fig. 3A for the location of *fabA* primer binding sites). To compare *fabA* transcription levels from promoters located in far upstream or *PA1611-fabA* intergenic sequences, βGal activities were measured in *lacZ* fusion strains harboring the putative *PA1612*-*PA1611* operon promoter, the entire *PA1612-PA1611* operon and the *PA1611-fabA* intergenic sequence (*PA1612′-lacZ*) or only the *PA1611-fabA* intergenic region (p1′-*lacZ* or p2′-*lacZ*) (Fig. 3D). It is evident that βGal levels expressed from *PA1612*′-*lacZ* were two times higher than those expressed from p1′-*lacZ* or p2′-*lacZ* (Fig. 3D). Also, predictions of transcription terminators within *PA1613* through *fabB* using TransTerm software (http://cbcb.umd.edu/software/transterm/ttgenbank50/Pseudomonas aeruginosa.tt) indicated that there are no putative transcriptional terminators in the *PA1612* through *fabB* interval, but only downstream of *fabB*. In summary, both RT-PCR and gene fusion analyses suggested that *fabA* may be co-transcribed with *PA1612-PA1611* or another promoter located outside of the *PA1611*-*fabA* intergenic region. This probably explains why identification of transcription start sites using primers with binding sites in the *fabA* 5′ region failed (data not shown). Similarly, other research group determined that the *fabA* mRNA transcribed by another promoter located outside of the *PA1611*-*fabA* intergenic region was identified and seemed to be the most abundant compared to the shorter transcript using 5′-RACE and Northern blot analysis [22].

### Negative regulators of *fabAB* expression

**Figure 4:**
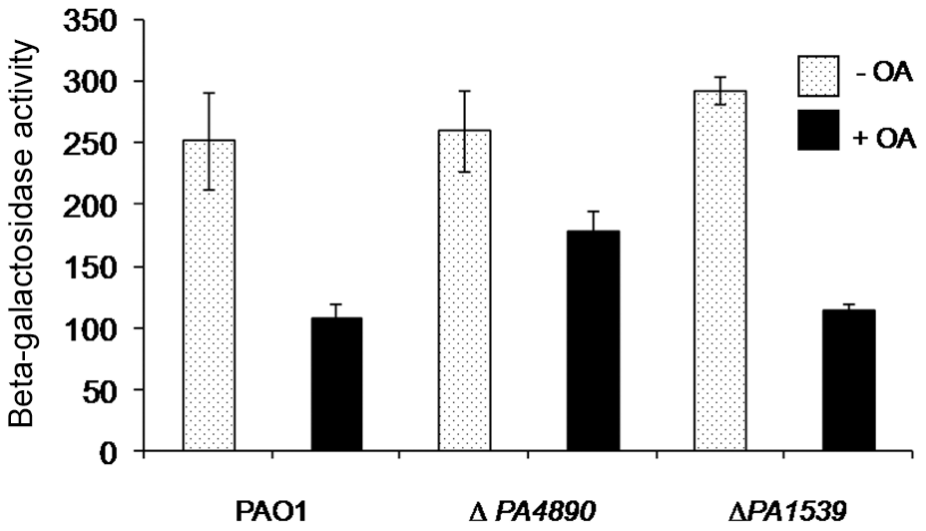
Effects of FabR homologs on *fabA*′-*lacZ* expression. β-galactosidase activities were measured in wild-type PAO1 and Δ*PA4890* and Δ*PA1539* mutant strains containing a chromosomal *fabA*′-*lacZ* fusion. Cells were grown in LB medium. Where indicated, 0.05% oleic acid (OA) was added to cells with 0.05% Brij-58.

Besides FadR, an additional regulator of *fabA* and *fabB* gene expression in *E. coli* was originally suggested by a computational footprinting method. An actual binding of this “virtual” regulator was subsequently confirmed using DNA affinity chromatography [9], [21]. Later, FabR (YijC) was found to function as a repressor of *fabB* gene expression [9]. *P. aeruginosa* has two FabR homologs, *PA4890* and *PA1539*, which have about 50% sequence similarity to *E. coli* FabR. To assess whether these homologs repress *fabAB* expression in *P. aeruginosa*, β-galactosidase activities were measured in wild-type, Δ*PA4890* and Δ*PA1539* mutant strains containing a chromosomally-integrated *fabA*′-*lacZ* fusion. The addition of OA repressed *fabA* transcription in all three strains tested (Fig. 4). In the Δ*PA4890* strain, however, OA repressed *fabA* expression to a lesser extent than in wild-type and the Δ*PA1539* strain. However, deletion of *PA4890* did not affect *fabA* expression in the absence of OA, indicating that only PA4890 is involved in negative regulation of UFA synthesis in the presence of OA. In addition, gel shift assays utilizing purified protein showed that PA4890 binds to the putative FabR-binding site located upstream of *fabA* [22]. Later, PA4890 was renamed DesT, a repressor *desBC* operon expression. Our data suggest that DesT may also negatively regulate UFA synthesis via regulation of *fabAB* operon expression.

### Positive regulation of *fabAB* expression

Our previous studies indicated that expression of the *fabAB* operon was up-regulated in biofilm-grown cells, probably because in biofilms bacteria grow under anaerobic conditions (data not shown). Furthermore, *lacZ* expression in a wild-type strain containing a chromosomally integrated *fabA′-lacZ* fusion was repressed by 40%–50% in the presence of 0.05% of OA (Fig. 2). In other words, the addition of exogenous UFA resulted in repression of *fabAB* transcription, indicating that its expression may be regulated by unknown activating factors. This situation is reminiscent of the one found in *E. coli* where in the presence of exogenous UFAs FadR binds acyl-CoAs and the acyl-CoA-FadR complex then binds to its cognate binding site, resulting in repression of *fabA* transcription. These results suggested the existence of a FadR-like *fabAB* regulatory gene in *P. aeruginosa*.

### Deletion of candidates to identify putative regulatory genes

*PA1611* was considered as a candidate for a *fabAB* regulatory gene, because of its location upstream of *fabA* and because it encodes a two-component regulatory system (a probable sensor kinase/response regulator hybrid) and thus, may sense exogenous UFAs similar to what has been demonstrated in *B. subtilis* and other Gram-positive bacteria. However, deletion of *PA1611* had no effect on expression of a *fabA*′-*lacZ* fusion (data not shown). The *PA1627* gene product is 26% homologous to *E. coli* FadR, which is known to positively regulate UFA synthesis, but deletion of *PA1627* had no effect on transcription of a *fabA*′-*lacZ* fusion (data not shown). Although UFA synthesis is increased when cells are grown under low temperature growth conditions in several bacteria, including *E. coli*, *PA1611* and *PA1627* deletion mutants grown at 16°C had similar levels of *fabA* transcription when compared to wild-type PAO1 (data not shown). Taken together, it can be concluded that neither *PA1611* nor *PA1627* are involved in regulation of *fabAB* transcription. Previously, all unidentified transcriptional regulators of the GntR family were deleted in PAO1 containing a chromosomally inserted *fabA*′-*lacZ* fusion by employing a high-throughput Gateway cloning technology [10]. Significant changes in *lacZ* expression levels in those knockout mutants would be indicative of positive regulation of *fabAB* transcription. However, none of the mutations did affect *fabA* expression and, therefore, transcription factors of the GntR family do not seem to be involved in the regulation of *fabAB* expression in cells grown either at 37°C or 16°C.

### *mariner*-based random insertional mutagenesis of a *fabA*(Ts) strain to identify putative regulatory genes

*mariner*-based random mutagenesis of potential *fabAB* activators in strain PAO1 *fabA*(Ts), which is a conditional mutant requiring OA supplementation only at 42°C, will create unconditional mutants requiring OA supplementation at all temperatures. OA auxotrophs were identified by observing cell growth on LB without OA. Transposon insertions were transferred back to the *fabA*(Ts) strain to verify linkage between transposon insertion and the unconditional OA auxotrophy phenotype. Transposon insertion sites in mutants classified as OA auxotrophs were determined by nested PCR and DNA sequencing. *mariner* insertions causing OA auxotrophy due to inactivation of *fabAB* were excluded from further consideration. Several transposon insertions causing the desired phenotype were found and insertion sites were localized to the genes encoding the *σ^54^* factor *rpoN*; *PA0286* (*desA*), encoding aerobic fatty acid desaturase; *PA1629*, encoding a probable enoyl-CoA hydratase/isomerase; *PA4760*, encoding a putative heat shock protein; *PA4233*, encoding a probable major facilitator superfamily (MFS) transporter; and several other genes – e.g. *PA0358*, *PA3649*, and *PA4476* - encoding proteins of unknown function (Table 3).

**Table 3:** *mariner* insertions in genes causing unconditional UFA auxotrophy in *fabA*(Ts) PAO1.

Mutant	Gene with *Mariner* (TnM) insertion	Function
# 6	*rpoN*::Tn*M*	σ^54^ transcription factor
# 16	*PA4760*::Tn*M*	Heat shock protein DnaJ
# 25	*PA1629*::Tn*M*	Probable enoyl-CoA hydratase/isomerase
# 30	*PA0358*::Tn*M*	Hypothetical protein
# 39	*PA3649*::Tn*M*	Hypothetical protein (63% similar to *E. coli yaeL*)
# 44	*PA4476*::Tn*M*	Hypothetical protein
# 75	*PA4233*::Tn*M*	Probable major facilitator superfamily (MFS) transporter
# 77	*PA0286* (*desA*)::Tn*M*	Fatty acid desaturase DesA

To assess whether any given mutation generated by *mariner* transposon insertion directly affected *fabAB* expression, chromosomal DNA fragments of Gm^r^ auxotrophic integrants were transferred into the parental strain containing a chromosomally integrated *fabA*′-*lacZ* fusion and βGal activities were measured in cells grown in the presence of OA. These experiments showed that all transformants had similar levels of *fabA* expression when compared to the wild-type, indicating that the products of the mutated genes affected *fabAB* expression indirectly (data not shown). These data also indicated that a considerable number of genes and metabolic pathways seem to be affect *fabAB* expression.

To directly screen for mutants affecting *fabA* transcription, the PAO1 *fabA*(Ts) strain containing a chromosomally integrated *fabA*′-*lacZ* fusion was used for *mariner*-based random mutagenesis. *mariner* insertion in an activator encoding gene would not only create an unconditional mutant requiring OA supplementation at all temperatures, but also result in lighter blue colonies caused by decreased *fabA*′-*lacZ* expression. A significant decrease in βGal activities was observed in 14 mutants among the 62 OA auxotrophs that were identified in this screen. Among them, only three mutants were identified as ones contained *mariner* insertions in the *PA2222*-*PA2223* locus encoding hypothetical protein and hypothetical outer membrane protein by nested PCR amplification and sequencing (data not shown). However, since the *PA2222-PA2223* operon could not be deleted, a further characterization of this operon was not pursued. However, for future studies transposon-generated *PA2222-PA2223* mutants could be employed to further study the role of these genes in *fabAB* regulation.

### Use of a surrogate host strain to identify potential *fabAB* regulators

**Figure 5:**
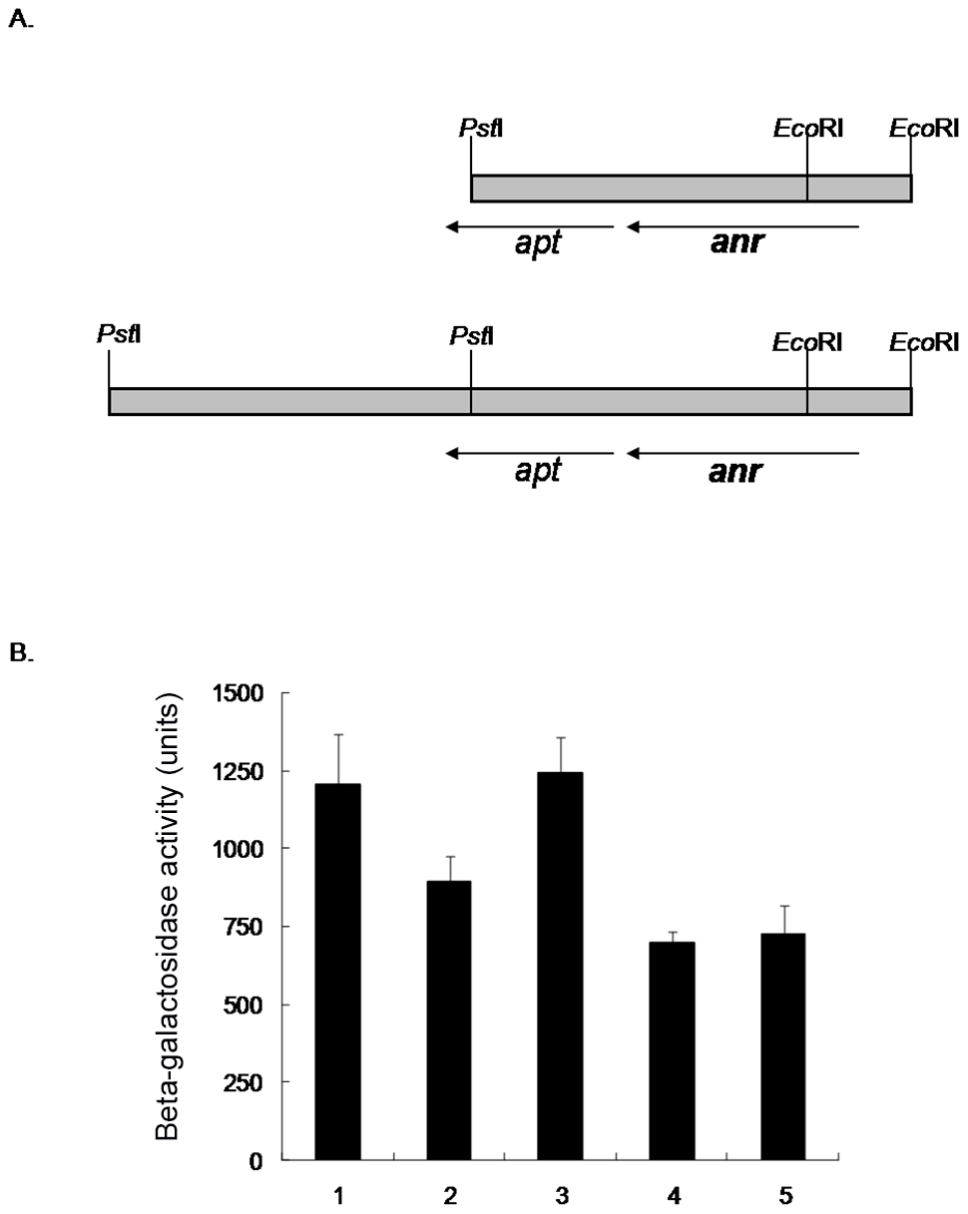
Effect of Anr in regulation of *fabAB* expression. **A**) *anr* is responsible for activation of *fabA*′-′*lacZY* transcription in *E. coli*. The restriction maps shown here are from cloned chromosomal DNA fragments isolated from blue, putative activator-expressing colonies. Plasmids isolated from ten blue colonies were sequenced by using M13 forward and reverse primers to verify the presence of the indicated genes. **B**) Effects of *anr* on *fabA* expression in *P. aeruginosa*. β-galactosidase activities were measured in wild-type and Δ*anr* PAO mutants containing a chromosomally integrated p*fabA*′-*lacZ* fusion. Columns: 1, PAO1 (wild-type); 2, PAO1010 (PAO1 Δ*anr*); 3, PAO1010 with pPS1684 (pUCP20 with *anr* ^+^); 4, PAO1010 with pPS1682 (pVLT35 with *anr*+) in the absence of IPTG; 5, PAO1010 with pPS1682 in the presence of IPTG.

The complex regulation of *fabAB* operon expression in *P. aeruginosa* may hamper the identification of directly acting *fabAB* activator(s) by genetic means. To avoid this complexity and interference with other factors, an attempt was made to identify activating factors in a Δ*lac E. coli* strain containing a chromosomally-integrated *fabA′-′lacZY* translational fusion. This experimental approach is illustrated in Fig. 1. A genomic library of *P. aeruginosa* PAO1 was transformed into the *E. coli* strain with the *fabA′-′lacZY* gene fusion. Potential activators would stimulate *fabA′-′lacZ* expression and transformants encoding such factors were therefore selected as dark blue colonies on X-Gal indicator medium. Linkage of this phenotype in transformants to plasmids encoding activating factors was confirmed by re-transforming the plasmids into the same *E. coli* host cells. Twenty-one isolates exhibited a blue colony phenotype. Restriction analyses of plasmids isolated from these transformants indicated the presence of the same *Pst*I-*Eco*RI fragment, but different flanking fragments. Besides the 2.9 kb fragment of pUC18 vector, the restriction enzymes released 0.4 kb, 1.2 kb, and 2.5 kb fragments. Among these, the 0.4 kb and 1.2 kb fragment were present in all plasmids, indicating that this region is responsible for activating *fabA* expression. These plasmids were classified into two groups and one plasmid from each group was selected for sequencing by using M13 forward and reverse primers. Two types of inserts were identified, those shown in the upper part of Fig. 5A (50%) and those shown in the lower part of the figure (50%). The only intact gene contained on both types of plasmids was *anr* (anaerobic NO_3_^−^ regulator), which presumably activated *fabA′-′lacZY* transcription. To assess a possible role of Anr in regulation of *fabAB* expression in *P. aeruginosa*, the *anr* gene was deleted from PAO1 containing a chromosomal p*fabA*′-*lacZ* fusion and βGal activities were measured in wild-type PAO1 and its Δ*anr* mutant. The presence of the *anr* deletion reduced *lacZ* expression only by ∼25% compared to the wild-type (Fig. 5B). In addition, this effect could only be complemented when *anr* ^+^ was present on the high-copy number plasmid pUCP20, but not on the low-copy number plasmid pVLT35, even with IPTG induction (Fig. 5B).

### Biochemical attempts aimed at identification of *fabAB* regulatory proteins

Since previous experimentation suggested that the 30 bp sequence within the *fabA* upstream region has a regulatory function in *fabAB* expression, fragments containing this sequence were used for identification of DNA binding proteins. Affinity chromatography strategies using streptavidin beads and DIG failed to obtain 30 bp-bound protein on SDS-PAGE gel. When CNBr-activated Sepharose beads were used as a ligand to immobilize DNA binding protein, distinct band on SDS-PAGE gel was successfully collected, subjected to in-gel trypsin digestion, and peptide mass fingerprinting. However, there was no good matched protein. Unfortunately, none of the strategies were successful in identifying regulatory proteins, indicating that the experimental conditions employed in these studies were possibly not correct. *fabA* expression is highly upregulated in biofilm-type grown cells, suggesting that anaerobic growth or biofilm-type growth might be proper conditions for identification of *fabAB* regulatory proteins.

### Analysis of a number of stable secondary structures of *fabA* 5′ untranslated region (UTR)

**Figure 6:**
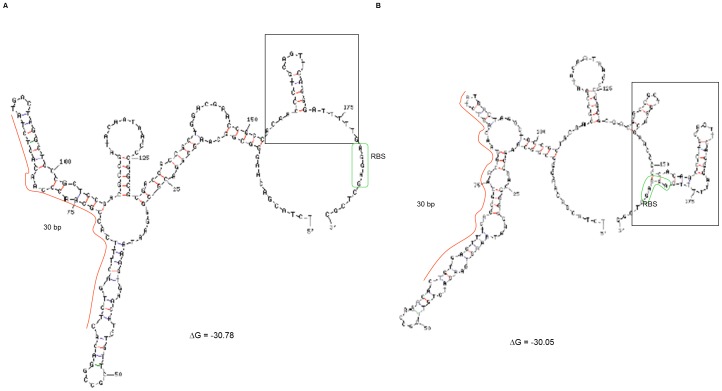
Secondary structures of the *fabA* 5′ untranslated region (UTR) by DINAMelt analysis. **A**) Terminator-containing structure. **B**) Antiterminator-containing structure. The red and blue lines indicate G-C and A-T complementary hydrogen bonds, respectively. The green and black boxes indicate ribosome-binding site (RBS) and terminator, respectively.

DINAMelt analysis (http://www.bioinfo.rpi.edu/applications/hybrid/hybrid2.php) of the *fabA* 5′ untranslated region (UTR) revealed that this region can assume a number of stable secondary structures. Two of these structures are shown in Fig. 6. These structures exhibit some of the criteria of riboregulatory elements [23]: i) they are found in the 5′ UTR; ii) they contain a number of stem-loop structures, one of which is conserved for ligand binding and the others are variable. It is noteworthy that the structure shown in Fig. 6A contains a stem-loop followed by a stretch of U residues, a situation found in typical *rho*-independent transcriptional terminators. This stem-loop structure is not present in the second structure shown in Fig. 6B.

## Discussion

Previous work demonstrated that UFA biosynthesis in *P. aeruginosa* is governed by two pathways, depending on the oxygen availability: 1) two aerobic fatty acid desaturase pathways consisting of DesA and DesBC, and 2) the anaerobic FabAB pathway. The *fabAB*-encoded proteins play an essential and dominant role in the UFA biosynthesis under both aerobic and anaerobic conditions.

While the existence of and the mechanisms of UFA synthesis via the anaerobic pathway were known at the onset of the studies presented here, little was known about the regulation of expression of the *fabA* and *fabB* genes. It was known that, unlike *E. coli* where *fabA* and *fabB* map to two distinct locations on the chromosome, these two genes form an operon in *P. aeruginosa*. It was also known that the expression of this operon was up-regulated in biofilm-grown cells and repressed by addition of fatty acids, especially oleic acid, to the growth medium. While this study did not reveal any specific regulatory protein(s) involved in regulation of *fabAB* operon expression or the exact molecular mechanisms governing regulation of expression of this operon, they revealed some previously unknown findings.

First, RT-PCR analyses indicated that the *fabAB* operon is transcribed from at least two promoters. It is co-transcribed with the upstream, seemingly unrelated *PA1612-PA1611* operon, but an additional promoter located in the *PA1611-fabA* intergenic region which also contributes to *fabAB* expression.

Second, the DesT (FabR) repressor binds to a palindromic sequence in the *PA1611-fabA* intergenic region, but seems to play only a modest role in the *fabAB* operon expression.

Third, a 30 bp sequence present in the *PA1611-fabA* intergenic region is a regulatory element involving the positive regulation of *fabA*. The position of this sequence at an appropriate position upstream of a putative -10 promoter consensus sequence indicates that it may be an activator-binding site.

Fourth, while *E. coli* FadR belongs to the GntR family of transcriptional regulators, deletion of none of the 25 genes encoding the GntR family of regulators in *P. aeruginosa* affected *fabA* transcription. Therefore, the *P. aeruginosa* FadR-like activator, if it exists, must belong to a different family of regulators. Additionally, deletion of the immediate upstream gene *PA1611* encoding a hybrid sensor kinase/response regulator protein did not adversely affect *fabA* transcription, at least not under the conditions employed in the present studies.

Fifth, numerous other cellular factors including RpoN, DesA and Anr seem to play at least minor roles in regulation of *fabAB* transcription, possibly through modulation of intracellular fatty acid levels or other metabolites. RpoN is a global regulator which regulates expression of nitrogen assimilation gene and fermentation gene expression (reviewed in [24]). However, there is no evidence that *rpoN* regulates UFA biosynthesis in *P. aeruginosa*. If it does, it may be due to global effects. According to previous study, *PA0286* was shown to encode DesA, an aerobic fatty acid desaturase [3]. This gene might be closely linked to the regulation of the *fabAB* operon by affecting cellular UFA levels. Enoyl-CoA hydratase/isomerases are involved in fatty acid metabolism. These hydratase activities catalyze the hydratation of 2-*trans*-enoyl-CoA into 3-hydroxyacyl-CoA and the isomerase activities shift the 3- double bond of the intermediates of UFA oxidation to the 2-*trans* position. The *anr* gene product, which senses low oxygen, supports anaerobic growth by activating numerous genes. *P. aeruginosa* is able to survive in an anaerobic environment. Since *P. aeruginosa* grows as a biofilm-type in the anaerobic CF lung mucus, anaerobic conditions may be an important factor to regulate metabolic pathways for robust growth and establishment of persistent infections. The anaerobic survival mode is supported by denitrification of nitrate or nitrite [25]-[27]. In addition, the arginine deaminase (ADI) pathway plays a key role in catabolizing L-arginine to L-ornithine, with the formation of ATP from ADP, resulting in the growth of *P. aeruginosa* under anaerobic condition in the absence of terminal electron acceptors such as molecular oxygen or nitrate (reviewed in [28]). Since Anr globally regulates expression of many genes, *P. aeruginosa fabAB* regulation via Anr may be indirect via other Anr-dependent factors. An alternate explanation might be that Anr activity may be inhibited by another factor(s) which may be highly expressed under aerobic growth, but Anr expression from a high-copy number plasmid may be able to saturate this “anti-Anr” factor and thus complement the deletion mutant. Since Δ*anr* mutants are unable to grow anaerobically, additional experiments should be performed with cells grown under microaerophilic conditions to further assess the potential role of Anr in *fabAB* gene expression. In conclusion, use of gene fusion technology revealed Anr as an activator of *fabA′-lacZ* expression in *E. coli*, but it played only a minor and probably indirect role in *P. aeruginosa*. While none of these findings does yet provide a clear picture of the molecular mechanisms governing transcription of the *fabAB* operon, they indicate that regulation of *P. aeruginosa fabAB* operon expression is very complex and most likely quite different from what has been described in *E. coli*.

Sixth, DINAMelt analysis suggests that *fabAB* expression may be regulated not via protein-binding, but via a yet-to-be discovered mechanism. One possibility lies in small regulatory RNA (srRNA)-mediated regulation of *fabAB* expression. srRNA can regulate gene expression at the transcriptional and translational levels. Intracellular metabolites such as amino acids, sugars and nucleotides can bind to *cis*-acting metabolite-sensing regulatory RNA elements and control gene expression, which are called riboswitches (reviewed in [29]). Various types of riboswitches are present in bacteria. Generally, non-coding regulatory RNA elements are found in the 5′-untranslated region (5′-UTR) and can give rise to three-dimensional conformational alternate changes in response to changes intracellular metabolite signals. Recently, it was found that besides metabolites, metals such as intracellular Mg^2+^ can regulate the Mg^2+^ transporter MgtA of *Salmonella enterica* serovar Typhimurium by the metal-sensing 5′-UTR of the *mgtA* gene [29]. Furthermore, changes in environmental conditions such as temperature can result in a conformational change of regulatory RNA, thus functioning as a “thermometer”. For example, virulence genes are highly expressed at 37°C by a PrfA transcriptional activator, which is thermally regulated by the 5′UTR of mRNAs of *prfA* in *Listeria monocytogenes* [30]. In addition, the ROSE (repressor of heat-shock gene expression) element placed in the 5′UTR region of heat-shock genes in many Gram-negative bacteria senses temperature changes in order to control their gene expression [31]. *E. coli rpoS* mRNA translation is regulated by the DsrA small RNA which disrupts a sequestering helix of a ribosome binding site during temperature downshift [32].

*P. aeruginosa* may employ a regulatory RNA for controlling anaerobic UFA synthesis. One probable pathway is that a metabolite-sensing non-coding RNA may recognize intracellular or exogenous UFA and change its structure, resulting in *fabAB* gene repression. This is consistent with the observation that OA supplementation repressed *fabA* expression up to 50% (Fig. 2). Another possible explanation is that low growth temperature might be an important controlling factor. Since cells should maintain the membrane fluidity for normal function required for transport and movement, they adapt to changes in environmental conditions, especially temperature. Upon exposure to low temperatures, cell membranes become solid-state, but they preserve their fluidity by increasing UFA levels. According to previous study, *fabA* expression is highly up-regulated in cells grown at 16°C compared to cells grown at at 37°C, suggesting positive regulation by low temperature [14]. Therefore, the 5′-UTR of *fabA* may sense low temperature and coordinate an adaptation process caused by a temperature downshift. However, having said all of this, searches of the *fabAB* upstream sequences have yet to reveal possible srRNAs.

**Figure 7:**
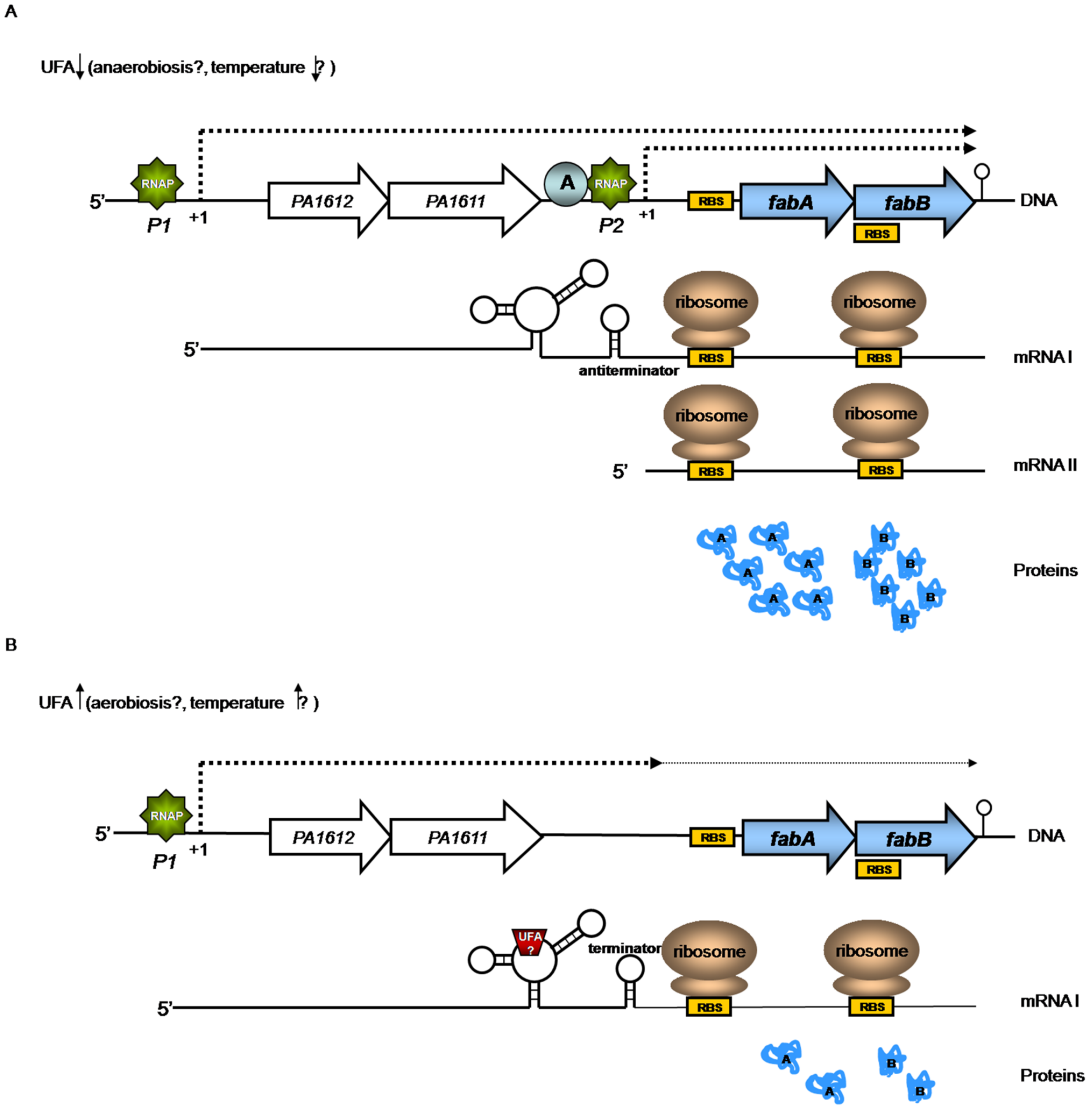
A working model for *P. aeruginosa fabAB* regulation.

As mentioned above, the *fabAB* operon is transcribed from at least two promoters, P1 and P2, resulting in transcription of two mRNAs, mRNA I and mRNA II (Fig. 7). In the absence of exogenous UFAs and under anaerobic, as well as perhaps low temperature conditions, the *fabAB* operon is transcribed from both of these promoters and the respective transcripts terminate at a single transcriptional terminator which is located immediately downstream of the *fabAB* operon (Fig. 7A). Transcription from P2 requires an activator protein which binds to the 30 bp region. Maximal transcription and translation ensures an adequate supply of FabA and FabB proteins for UFA synthesis. At the mRNA level, this situation is characterized by a riboregulatory element configuration on mRNA I containing an antitermination element.

In the presence of exogenous UFAs and/or aerobic or high (37°C) temperature conditions, the proposed activator probably binds an UFA-CoA ligand and the activator-UFA-CoA complex dissociates from the 30 bp activation element resulting in loss or of *fabAB* transcription from P2 (Fig. 7B). At the mRNA level, this situation is characterized by a riboregulatory element configuration that contains a terminator. The result is a low level transcription and translation of *fabA* and *fabB*, a desirable situation when oxygen levels and temperatures are high, exogenous UFAs are available and the overall cellular demand for UFAs is either low or can be satisfied by the activities of the aerobic desaturase pathways.

While many of our experimental results support this model, several questions do remain unanswered. For example, why were repeated genetic and biochemical attempts aimed at identification of the proposed activator protein unsuccessful? Why does purified DesT bind to the *PA1611*-*fabA* intergenic region, but there is little to no effect on *fabA* expression in *lacZ* fusion or microarray experiments? A possible explanation for this may be that most of the experiments described in this study were performed with cells grown aerobically and at 37°C, possibly conditions during which synthesis and/or activity of an activator protein is repressed or DesT activity is masked. This notion is supported by the fact that *fabA′-lacZ* expression was significantly higher in cells grown at 16°C when compared to cells grown at 37°C. Future experiments should therefore be performed with cells grown under anaerobic or microaerophilic conditions, as well as with cells grown at lower temperatures. Although DINAMelt analysis suggests the possibility that a riboregulatory element may be involved in regulation of *fabAB* operon expression, there is currently no experimental evidence supporting this possibility. Future efforts should therefore also include experiments that would address the existence of a 5′ UTR RNA with a high degree of secondary structure. The experiments would consist of in-line probing [33], [34] and RNase H cleavage assays of RNA-DNA hybrids [35] in the presence and absence of substrate to determine if the structure not only exists, but also undergoes a conformational change upon addition of the substrate or changes in an environmental cue. The nature of the substrate(s) or environmental cue(s), of course, remains speculative. Substrates could be metabolites such as a UFA-CoAs and environmental cue signals such as low temperature or low oxygen levels.

Studies on anaerobic UFA synthesis by the *P. aeruginosa* FabAB pathway and identification of its regulatory signals are significant because many infections caused by this bacterium, especially cystic fibrosis lung infections, are biofilm-type infections during which this bacterium's physiology is adapted to an anaerobic lifestyle. Such studies have therefore the potential for discovering new targets and drugs for treatment of biofilm-type infections.

## Supporting Information

Figure S1 **The sequence of** ***fabA*** **-** ***PA1611*** **intergenic region.** The last four *PA1611* codons and first eight *fabA* codons are shown. The conserved 30 bp sequence is boxed. A putative −10 region with good homology to the TATAAT consensus is indicated in underlined bold-faced letters. The DesT binding site is marked inverted arrows.
(TIF)

Figure S2 **The** ***fabA*** **upstream sequences are conserved in the three** ***Pseudomonas*** **spp.,** ***P. aeruginosa*** **,** ***P. putida*** **, and** ***P. syringae*** **.** The *PA1611*, *PP4173* and *PSPT02212* genes encode conserved hybrid sensor kinase/response regulatory proteins. Also highly conserved is the shaded 30 bp sequence. A lollipop structure indicates the transcriptional terminators of the respective *fabAB* operons.
(TIF)

Figure S3 **FabR binding sites in the** ***fabA*** **or** ***fabB*** **upstream regions of several bacteria.** The sequences in the gray box indicate conserved FabR binding sites. *P.p.*, *P. putida*; *P.s.*, *P. syringae*; *P.a.*, *P. aeruginosa*; *E.c., E. coli*; *Y.p., Y. pestis*; *V.c., Vibrio cholerae*. Only the *P.a.* and *E.c.* sequences were experimentally shown to bind FabR (*E.c.*) and its *P.a.* homolog DesT.
(TIF)

Figure S4 **Locations of binding sites for** ***E. coli*** **FadR or** ***P. aeruginosa*** **FadR-like activator and FabR in the** ***E. coli fabA*** **and** ***P. aeruginosa*** **PAO1** ***fabAB*** **upstream regions.**
(TIF)
