# Supplementary figures and images for: Characterization of Molecular Mechanisms Controlling fabAB Transcription in Pseudomonas aeruginosa
Source: PLoS One. 2012 Oct 2;7(10):e45646. doi: 10.1371/journal.pone.0045646 (PMC3462791; doi:10.1371/journal.pone.0045646)

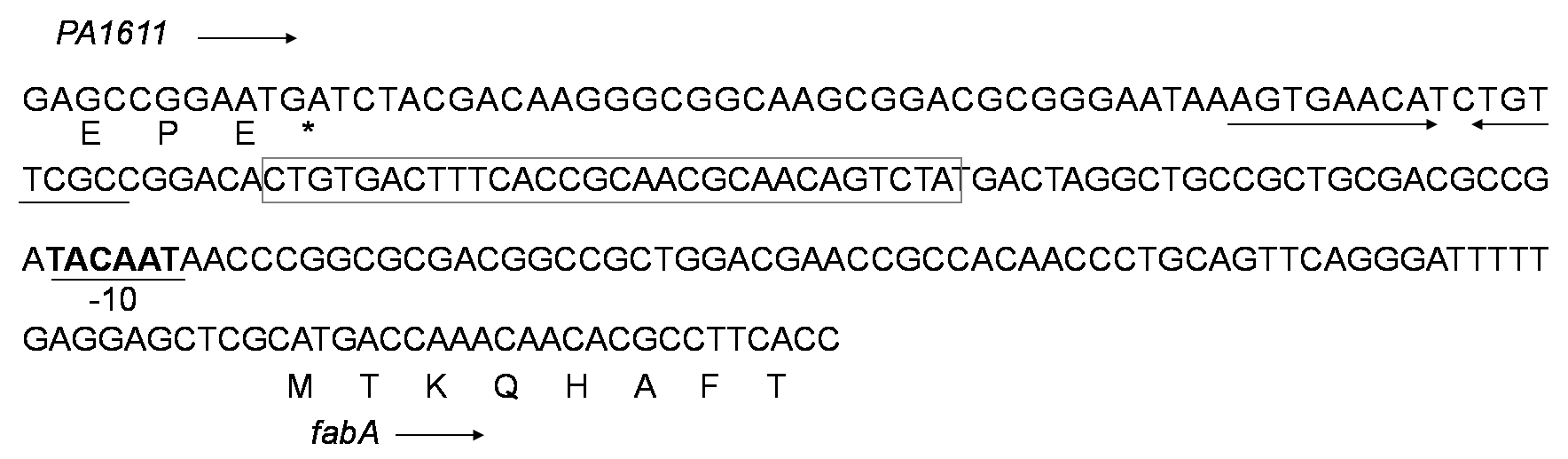

Supplement: Figure S1 — The sequence of fabA - PA1611 intergenic region. The last four PA1611 codons and first eight fabA codons are shown. The conserved 30 bp sequence is boxed. A putative −10 region with good homology to the TATAAT consensus is indicated in underlined bold-faced letters. The DesT binding site is marked inverted arrows. (TIF) [file pone.0045646.s001.tif]

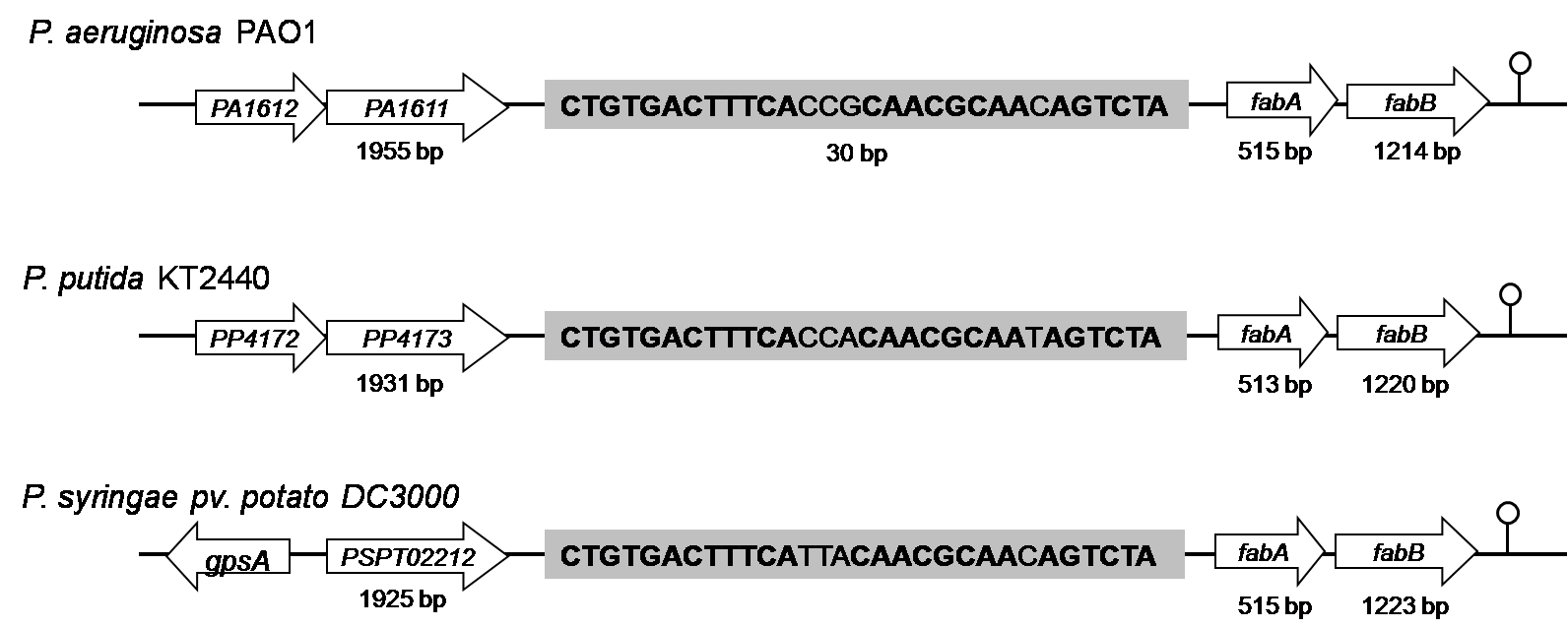

Supplement: Figure S2 — The fabA upstream sequences are conserved in the three Pseudomonas spp., P. aeruginosa , P. putida , and P. syringae . The PA1611, PP4173 and PSPT02212 genes encode conserved hybrid sensor kinase/response regulatory proteins. Also highly conserved is the shaded 30 bp sequence. A lollipop structure indicates the transcriptional terminators of the respective fabAB operons. (TIF) [file pone.0045646.s002.tif]

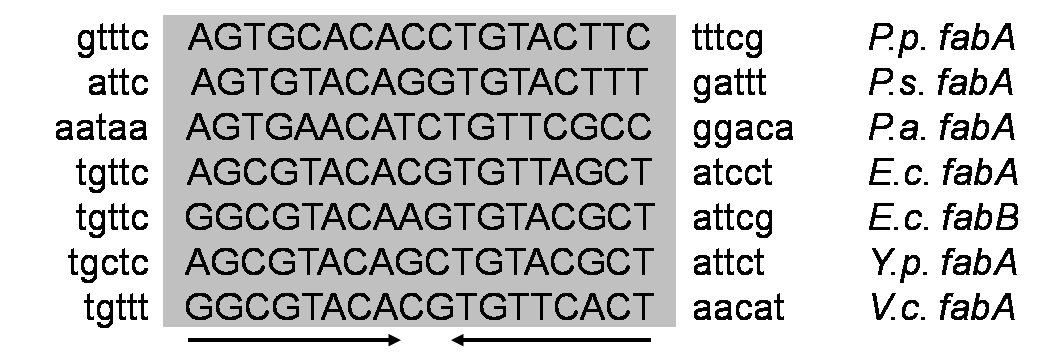

Supplement: Figure S3 — FabR binding sites in the fabA or fabB upstream regions of several bacteria. The sequences in the gray box indicate conserved FabR binding sites. P.p., P. putida; P.s., P. syringae; P.a., P. aeruginosa; E.c., E. coli; Y.p., Y. pestis; V.c., Vibrio cholerae. Only the P.a. and E.c. sequences were experimentally shown to bind FabR (E.c.) and its P.a. homolog DesT. (TIF) [file pone.0045646.s003.tif]

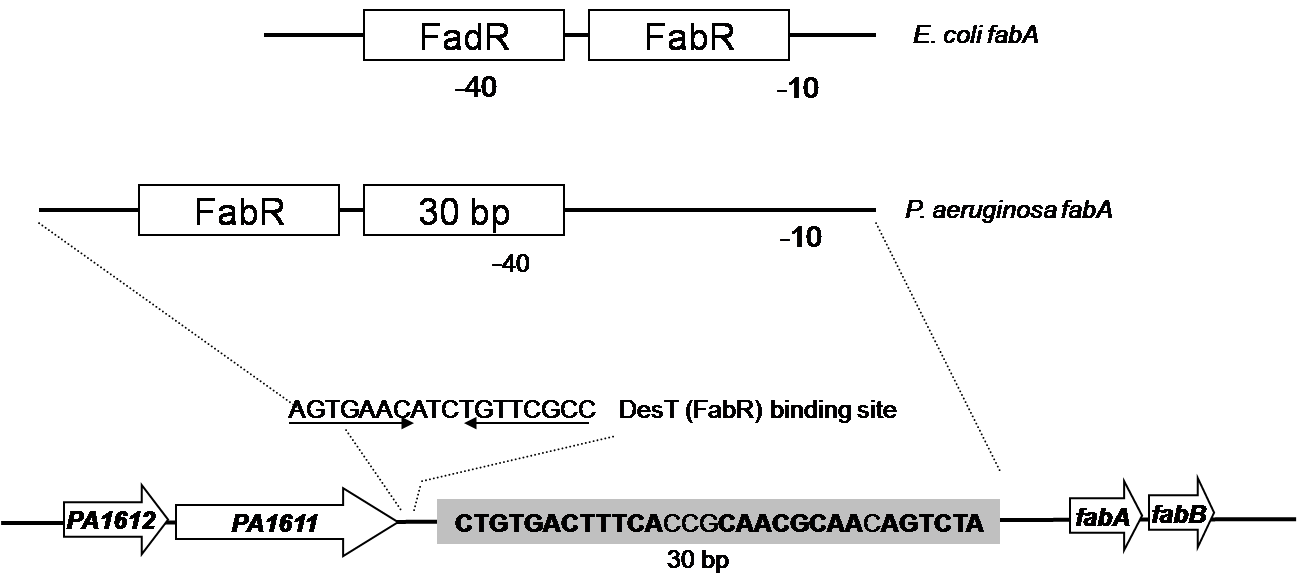

Supplement: Figure S4 — Locations of binding sites for E. coli FadR or P. aeruginosa FadR-like activator and FabR in the E. coli fabA and P. aeruginosa PAO1 fabAB upstream regions. (TIF) [file pone.0045646.s004.tif]
